# The detection and effect of social events on Wikipedia data-set for studying human preferences

**DOI:** 10.3389/fdata.2023.1077318

**Published:** 2023-03-30

**Authors:** Julien Assuied, Yérali Gandica

**Affiliations:** ^1^CY-Tech Cergy Paris Université, Cergy, France; ^2^Department of Mathematics & Master in Big Data, Universidad Internacional de Valencia (VIU), Valencia, Spain; ^3^CY Cergy Paris Universite, CNRS, Laboratoire de Physique Théorique et Modelisation, Cergy, France

**Keywords:** human preferences, Wikipedia, outliers detection, possible bias, massive events

## Abstract

Several studies have used Wikipedia (WP) data-set to analyse worldwide human preferences by languages. However, those studies could suffer from bias related to exceptional social circumstances. Any massive event promoting exceptional editions of WP can be defined as a source of bias. In this article, we follow a procedure for detecting outliers. Our study is based on 12 languages and 13 different categories. Our methodology defines a parameter, which is language-dependent instead of being externally fixed. We also study the presence of human cyclic behavior to evaluate apparent outliers. After our analysis, we found that the outliers in our data-set do not significantly affect the analysis of preferences by categories among different WP languages. While investigating the possibility of bias related to exceptional social circumstances is always a safe measure before doing any analysis on Big Data, we found that in the case of the first ten years of the Wikipedia data-set, outliers do not significantly affect using Wikipedia data-set as a digital footprint to analyse worldwide human preferences.

## Introduction

Editing the worldwide encyclopedia Wikipedia (WP) has several important implications. First of all, it is nowadays an essential and primary source of knowledge, accessible to everybody with access to the Internet. Secondly, that source of information does not follow an editorial line. Thus, different perspectives and points of view on the same subject can be read in an article if it is well-structured and democratized. The third point, which is the one that concerns us in this article, is that the information is a footprint of the community's cultural context. For example, by a cross-language analysis of over 40 languages, Miquel-Ribé et al. identified that at least a quarter of a WP page's content is generally dedicated to the specific cultural context (Miquel-Ribé and Laniado, 2018). That study confirms the inter-cultural nature of Wikipedia as a digital repository of human cultural context. In the same order of ideas, Mehler et al. (2020) have shown that information on a given topic is strongly language-dependent. Even the definition of the quality of an article depends on the WP language, as reported by Jemielniak and Wilamowski (2017). The presence of those important content imbalances among languages suggests the success of WP as a good proxy for analysing cultural context or group preferences.

In that respect, several studies regarding worldwide human attributes have been performed using the Wikipedia data-set. For example, Samoilenko et al. (2016) studied the similarity of interests between cultural communities and cross-lingual interconnections. In a previous work (Gandica, 2018), we studied worldwide human preferences showing, for example, which languages have uniform preferences among subjects, in contrast with the ones showing pronounced specific inclinations.

All those studies could suffer from some bias related to exceptional social circumstances, for example, special events, accidents; any conjuncture that can alter the regular pattern of editing WP based on self-choice. When using Wikipedia data-set for analysing human preferences, any massive event promoting the extraordinary edition of WP can be defined as a source of bias. In Data Science, the term for such bias is outliers. In general, outliers are data points that deviate markedly from the trend in the sample (Rousseeuw and Hubert, 2011). Those outliers could result from errors in the data, for example, errors during the encoding or even during the experimental setup. In that case, such points could be deleted from the data. However, outliers could also be a consequence of a considerable variance, in which case, it could point out some interesting phenomena.

An even more difficult situation is when the whole data-set presents bias due to massive actions, like special events or natural disasters. Then, if the scientific question is related to legitimate human preferences, those events could cause serious bias and cannot be detected as singular points due to their massive nature. In this work, we propose a methodology to deal with such a situation. Our methodology allows us to answer the question: Are the preferences between individuals sharing the same language, which is a trace of the collective identity of the entire group, the same whenever the effect of extraordinary events is removed?

The analysis has two parts. The first part deals with identifying outliers from monthly data points. Our methodology does not depend on any external threshold; the threshold depends intrinsically on each WP data-set. The second part aims to detect periodicity, and we use the Fourier transform for that endeavor.

Our study is based on the first ten years of Wikipedia data-set to avoid the artificial process of editing WP caused by the recommendation system that was applied by the Wikimedia Foundation to fill that gap caused by inner cultural preferences (Manske, 2019). Our analysis considers 12 languages: the ones written in Spanish (ES-WP), French (FR-WP), Portuguese (PT-WP), Italian (IT-WP), Hungarian (HU-WP), German (DE-WP), Russian (RU-WP), Arabic (AR-WP), Japanese (JA-WP), Chinese (ZH-WP), and Vietnamese (VI-WP). Our selection takes into account the interplay between a worldwide view and the WP sizes.

## The data-set

The activity of the first 10 years of WP editing activity WP was downloaded. The starting points depend on each WP language, and all the data-sets are in the range from 11/10/2001 to 28/03/2010. Bot activities were removed by detecting users whose name contains the word bot in either combination of both uppercase and lowercase. Table 1 shows the number of pages, users, and edits, and the starting date for each WP data set used in this study.

**Table 1:** Data-set.

**WP**	**No. of pages**	**No. of users**	**No. of edits**	**No. of starting date**
ES	1,144,177	2,682,095	47,728,243	24/09/2001–17:01:24
FR	2,936,383	203,038	58,325,545	13/10/2001–09:59:23
PT	894,521	1,475,236	19,937,771	17/06/2001–17:13:19
IT	1,084,333	97,161	22,200,807	14/09/2001–10:19:28
HU	248,808	148,068	5,758,998	09/07/2003–04:41:24
DE	1,111,265	357,561	39,689,676	09/09/2001–03:34:41
RU	1,134,752	76,066	14,199,590	13/11/2002–18:48:05
AR	624,118	23,641	7,674,946	11/07/2003–00:23:02
JA	690,795	126,657	24,584,471	10/09/2002–19:25:48
ZH	495,855	76,613	3,657,770	30/10/2002–17:19:19
VI	238,859	12,262	12,618,296	16/11/2002–14:54:24

In order to facilitate the comparison among preferences between different languages, we used the categories already classified in the main branch of the tree structure, defined by the Wikimedia Foundation itself, and found in https://en.wikipedia.org/w/index.php?title=special:categorytree&target=maintopicclassifications&mode=0&hideprefix=20&showcount=1. To avoid a substantial overlap between categories, we left out of this study the categories: Culture, Humanities, Law, Life, Matter, People, Reference Works, Science and Technology, Society, Universe, and World. Thus, our study is based on the 13 following categories: Arts, Sports, Right, Events, Philosophy, Geography, History, Games, Mathematics, Nature, Politics, Religion, and Health. We used the Petscan API^1^ in order to download the names of all the pages within each category in the 12 languages under study. Notice that different languages have different pages in the corresponding category.

## Trending

**Figure 1:**
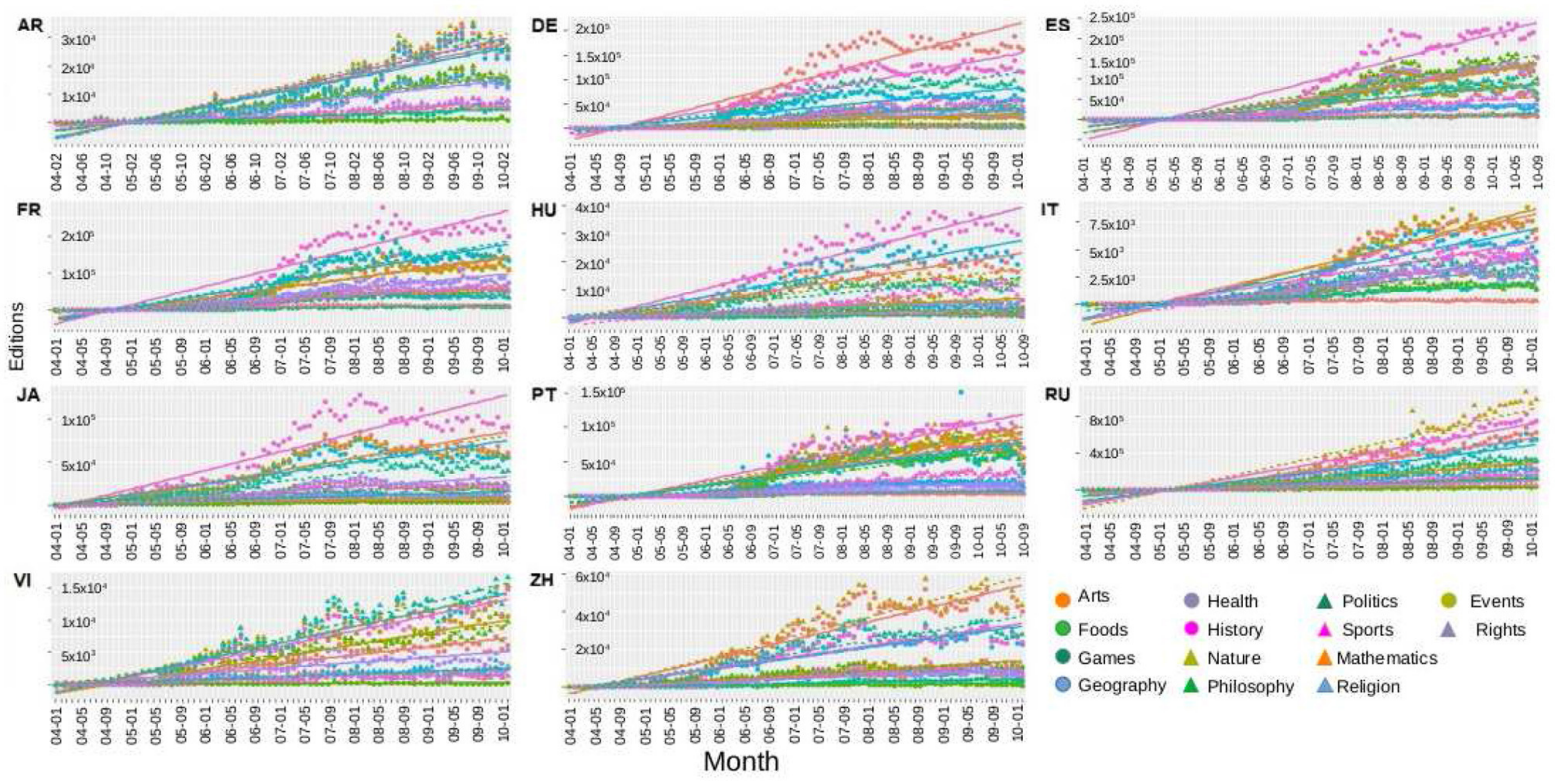
Trending: number of editions per months according to the categories, as represented by the legend. A trend line for each data set has been added. Each plot refers a different WP language. Three different behaviors have been identified, see text.

Let us start visualizing the data. In Figure 1, we show, for each language, the number of editions per month according to the categories. After playing with the axes, we realized that there was no power-law or exponential trending for the whole period in any curve. We got that points mostly move around linear trends in all the curves. For that reason, we have added a trend obtained from the linear regression to better visualize the behavior of the data for each category. However, let us notice that some curves present a cyclic behavior around those coarse-grained linear trends.

We can identify three different behaviors. Some data-set mostly follow their trends, as in most WP-RU categories. In some other cases, the points oscillate around the trends, as in WP-AR and WP-VI. Finally, some curves get far from the trend, such as History in WP-FR and WP-JA and Arts in WP-DE; however, they cross the linear trend several times.

We will analyse the last two cases separately. Outliers points will be located and eliminated from the data set where the curves are considerably separated from the trends. On the other hand, the case of oscillatory data will be inspected by employing a Fourier analysis, to detect the presence or not of periodicity.

## Finding outliers

We need to spot outliers for the data points that are far from the trend. For that endeavor, we first must define them. In order to decrease the bias caused by the researcher, we aim at fixing the threshold directly from the data rather than being externally fixed by the researcher. We hypothesize that during extraordinary massive events, extra editions are performed by regular editors, but also new editors enter information into WP. Then, extraordinary massive events will produce bias in both: the number of editions and users.

**Figure 2:**
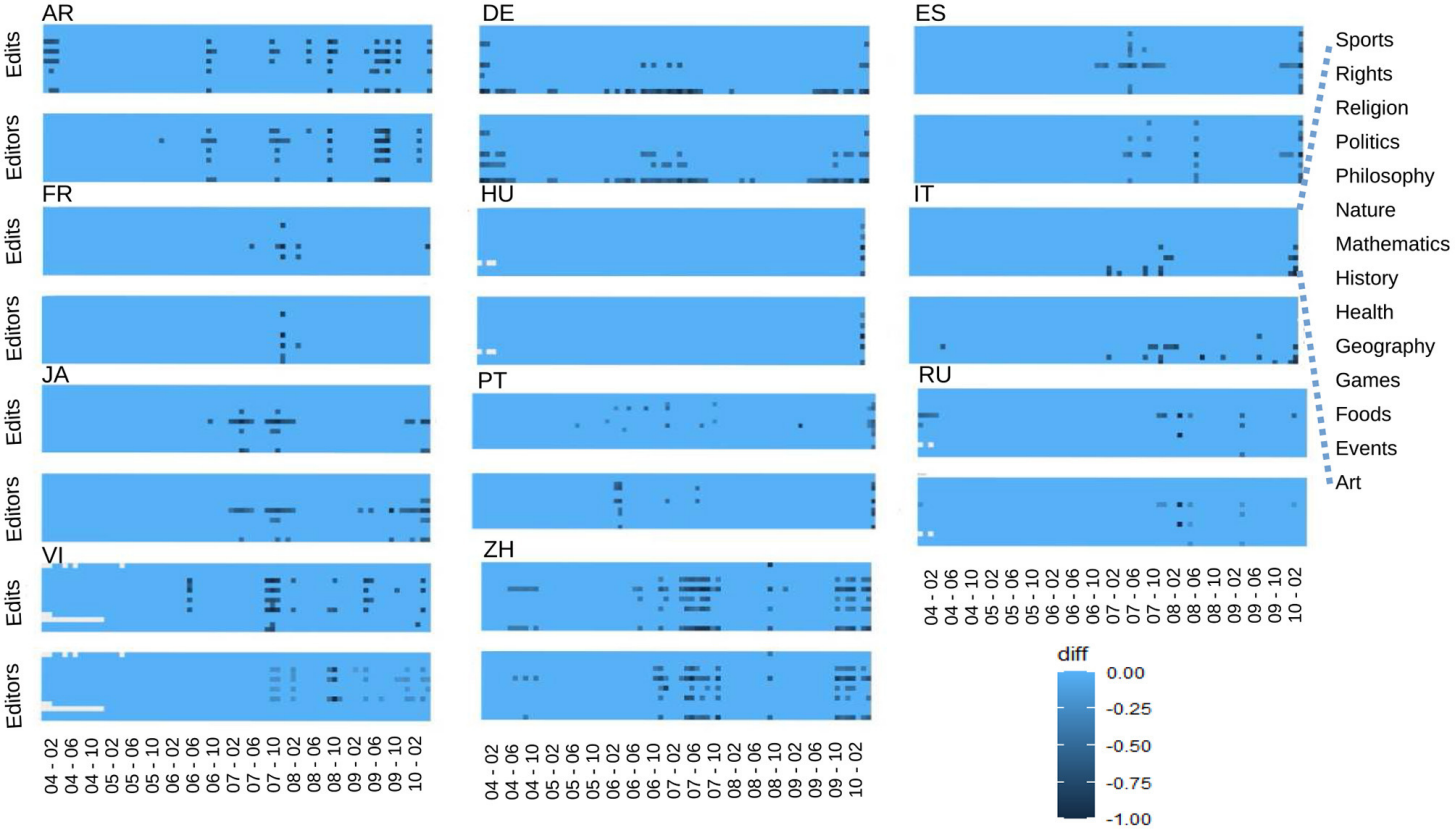
Finding outliers: Heat-maps showing the difference between the data points and trends for edits and editors, normalized in terms of the highest value of each graph. Each horizontal line is one category as signaled in the plot for Edits-IT. The heat-map legend is shown at the **bottom right**.

To fix a WP language-dependent threshold, we then start using the monthly time series for the number of edits (shown in Figure 1) and the monthly time series for the number of users. Next, the difference between the monthly points and their trend values are calculated for each category. We normalized the values of these differences in terms of the highest value of each graph. In this way, we generated, for each language, one plot for editors and one plot for editions, which are the heat-maps shown in Figure 2.

Next, for each language, we compared the two heat-maps: editors and editions. Finally, we took as the threshold the minimum value where those differences appear on both plots. All the data points whose difference with the trend is greater than that threshold value are defined as outliers. Let us notice that in Figure 2, each WP language has two plots. The one above (heat-map for edits) is the one which should be compared to Figure 1 (number of editions per month).

We have already identified outliers as the points that are far from the trends. However, we have seen that some curves present cyclic behavior. In fact, Wikipedia data-set presents cyclic patterns (Gandica et al., 2016). In other words, periodic behavior is intrinsic to human nature, and it can modulate the edition of Wikipedia. Thus, those cyclic points should not be identified as extraordinary situations; some examples are national days and commemoration of great tragedies. Following this order of ideas, if some data points were identified as outliers and resulted from intrinsic human cyclical patterns, we should not delete them from the data. The study of that cyclical behavior is performed in the next section.

## Cyclical analysis

**Figure 3:**
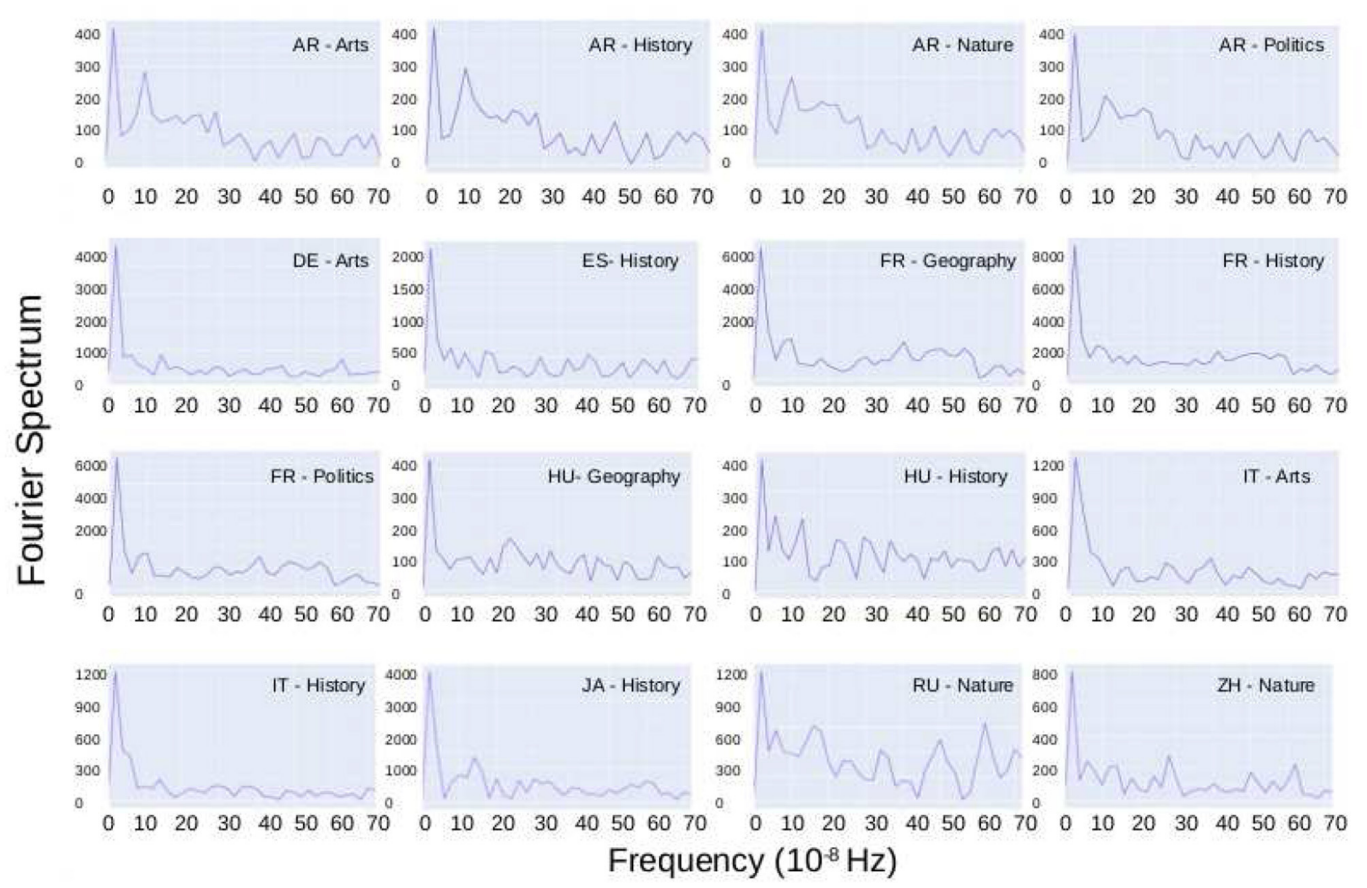
Cyclical analysis: Fast Fourier Transform for the temporal series where the amplitude is considerably large compared to the other categories of the same language. All the figures show a cyclic behavior of 12 months, and only the AR-WP presents a second pick for a period of 3 months.

As said before, some points distant from the trend show periodic behavior. As cyclical patterns are an essential feature of human activity, we will not consider them outliers. A natural way to study the periodicity of data is by using a Fourier power spectrum. In Figure 3, we show the Fast Fourier Transform (FFT) for the temporal series where the amplitude is considerably large compared to the other categories of the same language. All the figures show a cyclic behavior of 12 months (≈3.2 × 10^−8^*seg*^−1^). Only the AR-WP presents a second pick for a 3-months period (≈13 × 10^−8^*seg*^−1^). Notice that the input for the FFT is the original time series in seconds and not the data shown in Figure 1. We also found out daily periodicity; however, we do not report them because they are related to the expected daily human cycle, thus not interesting for the present study.

These signs of periodicity allow us to identify some apparent outliers on the heat-maps, which could have been considered as extraordinary events; however, their periodicity indicates that they are consequences of regular human periodic patterns (deeper than simple daily or weekly cycles).

## Results

After discarding cyclic behaviors as possible outliers, we identified two of the external events. The first one in January 2008 in Italy seems to have a link with an artistic event, then affecting the categories “Arts” and “Events.” The second event that we identified was in the Portuguese Wikipedia. We observed a peak of modification concerning pages related to geography and nature in October 2007. These articles follow the big fires in the Amazonian forest in Brazil, a Portuguese-speaking country.

To better identify all the external circumstances affecting the digital footprints of Wikipedia as a social repository to study human preferences by languages, we can see the top-edited WP pages; however, such an analysis is out of the scope of this work. On the contrary, our goal is to detect those events as outliers manifested by the same Big-Data analysis, i.e., letting the data speak.

**Figure 4:**
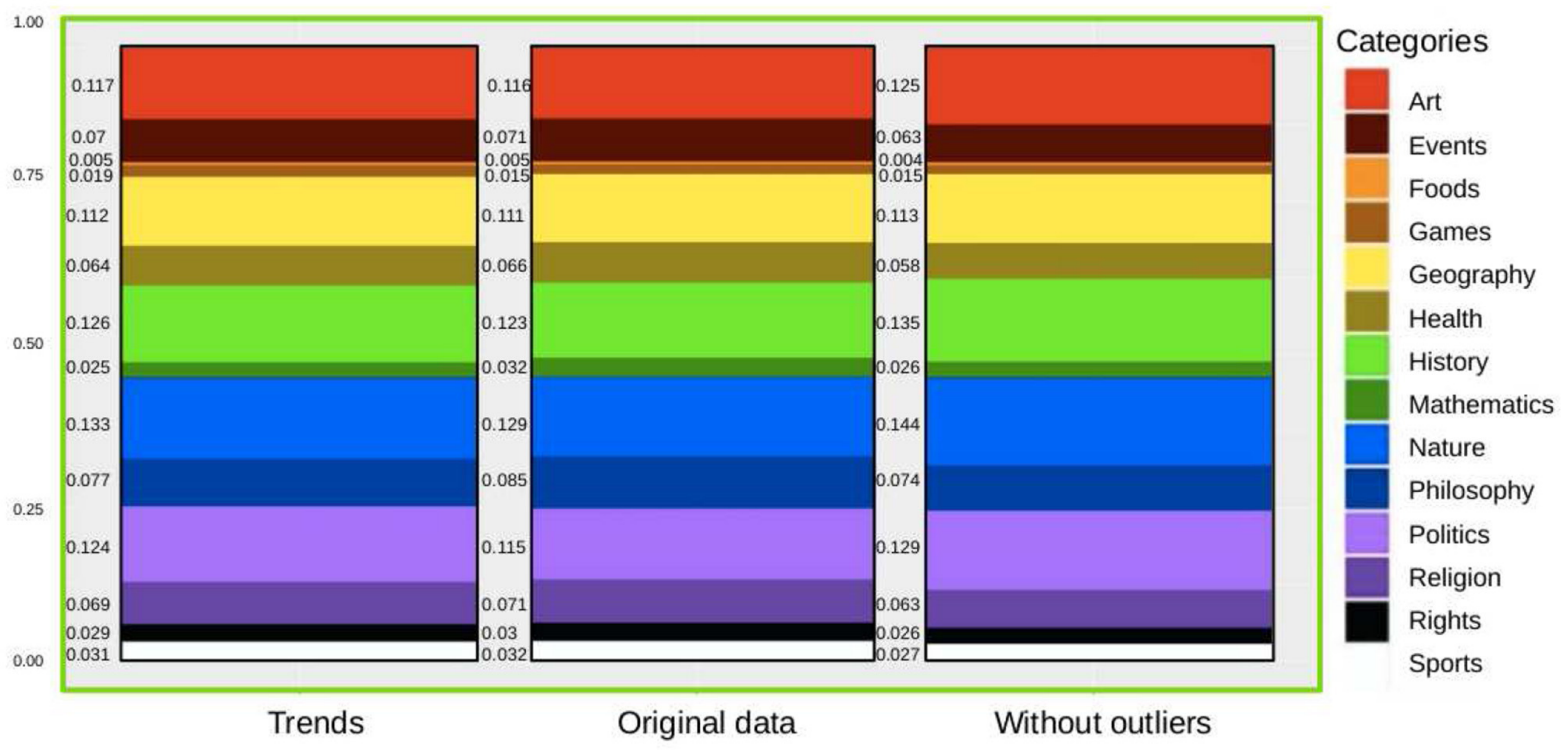
Comparison: normalized means of the ratio of categories per month on the VI-WP. We show the values for the trends, the original values, and the values without outliers.

Finally, in Figure 4, we show, as one of the examples, the proportions of the categories in the total sample of the Vietnamese Wikipedia (VI). We show the proportions for the trends, the original proportions (both shown in Figure 1), and also the proportions after removing the outliers, as explained throughout this manuscript. After computing the p-values of those data, we deduced that there is no significant difference, and the same happened for all the languages. In that respect, we show that the extraordinary events on the Wikipedia data-set are non-significant when using this data-set to analyse language preferences.

One of the problems characterizing the Wikipedia data-set is the presence of overlap between categories. We have diminished this problem by selecting a set of categories with few overlaps. However, slight overlaps still remain. Regarding the problem of finding outliers, as the study is performed in each category separately, then the detection of outliers in at least one category is enough to declare it as a real outlier of the data-set. Let us remember, as well, that our study covers the first 10 years of some languages in Wikipedia as a data-set.

## Conclusions

In this paper, we first explained the importance of detecting possible bias related to extraordinarily massive events when using any data set to analyse human patterns. Then, we develop a procedure to detect those possible outliers on the Wikipedia data set. Next, we analyzed cyclicality to separate outliers from typical human cyclical patterns, which should not be confused with atypical behaviors, namely outliers.

After processing the data, as explained above, we computed the proportions of categories as signs of preferences by language and found no significant difference.

Even though investigating the role of outliers is always a safe measure before performing any analysis in Big Data, at least in the case of the use of Wikipedia editions, during the first ten years (as we have used it here), the outliers do not have a significant effect on the results. A word of caution is worth mentioning, this work proposes a human-controlled methodology to detect outliers in Wikipedia data sets. There are several other data sets and other different ways to find outliers using, for example, Machine Learning tools. Here, we proposed a methodology that works well for Wikipedia data-set specifications, such as the editing process by regular users and cyclic behavior. Each data set has its characteristics. Consequently, it is necessary to reason on the data specifications to decide which methodology is best to apply.

## Data availability statement

Publicly available datasets were analyzed in this study. This data can be found at: http://konect.cc/.

## Author contributions

YG: conceptualization, formal analysis, methodology, and supervision. JA: software and visualization. YG and JA: data curation. Both authors contributed to the article and approved the submitted version.

## Footnotes

^1^Available online at: https://petscan.wmflabs.org/.
